# A systematic review and meta analysis on the effect of vitamin D in preeclampsia and gestational diabetes mellitus in pregnancy

**DOI:** 10.3934/publichealth.2025062

**Published:** 2025-12-18

**Authors:** Martha Irene Kartasurya, Tomasina Stacey, Naintina Lisnawati, Andi Rispah Sulistianingsih

**Affiliations:** 1 Department of Public Health Nutrition, Faculty of Public Health, Universitas Diponegoro, Indonesia; 2 Florence Nightingale Faculty of Nursing, Midwifery & Palliative Care, King's Collage London, 57 Waterloo Road, London, SE1 8WA, UK; 3 Doctoral Program of Public Health, Faculty of Public Health, Universitas Diponegoro, Indonesia

**Keywords:** vitamin D, preeclampsia, gestational diabetes, pregnancy, blood pressure, meta-analysis

## Abstract

**Background:**

Preeclampsia and gestational diabetes mellitus (GDM) are significant contributors to maternal and neonatal morbidity and mortality, particularly in low- and middle-income countries. Vitamin D might play a role in the pregnancy complication prevention. However, findings across studies remain inconsistent. In this review, we aimed to evaluate vitamin D deficiency effect on preeclampsia and GDM risks, and the effect of vitamin D supplementation during pregnancy in reducing preeclampsia and GDM incidences.

**Methods:**

We followed the PRISMA guidelines and registered the protocol with PROSPERO (CRD42024609276). Database PubMed, Scopus, and EBSCO-Medline were used to search cohort and randomized controlled trial (RCT) studies published between 1993 and 2025. Two reviewers independently assessed the article quality with the Joanna Briggs Institute checklists and extracted data. Meta-analyses were performed using RevMan 5.4. The results were reported in pooled odds ratios (OR) or standardized mean differences (SMD) with 95% confidence intervals.

**Results:**

A total of 52,372 participants from 24 studies were included in this review. Vitamin D supplementation appeared to reduce the preeclampsia risk by 42% (OR = 0.58; 95%CI: 0.43–0.78; I^2^ = 45%) and GDM by 45% (OR = 0.55; 95%CI: 0.36–0.87; I^2^ = 0%) in RCTs. Vitamin D supplementation is most effective in reducing the risk of recurrent preeclampsia in women with vitamin D deficiency. In cohort studies, vitamin D deficiency was associated with a higher risk of GDM (OR = 1.29; 95% CI: 1.16 to 1.43; I² = 7%), but was not significantly associated with preeclampsia (OR = 1.67; 95% CI: 0.92 to 3.01; I² = 85%).

**Conclusion:**

Vitamin D supplementation in pregnancy, especially in the first trimester, decreased preeclampsia and GDM risks, while vitamin D deficiency in pregnancy increased GDM risk but not preeclampsia. These findings support the potential benefit of vitamin D supplementation in the routine antenatal care to improve pregnancy outcomes.

## 1. Introduction

Pre-eclampsia occurs in about 3%–5% of pregnancies and is linked to an estimated 42,000 maternal deaths per year [1]. For every death caused by pre-eclampsia, between 50 and 100 women experience significant health complications [2]. Low- and middle-income countries (LMICs) face the most significant impact, as limited resources and restricted access to quality obstetric care and family planning services contribute to higher rates of serious complications compared to high-income nations [2]. The International Association of Diabetes and Pregnancy Study Groups (IADPSG), stated that 14.7% of pregnant women worldwide had gestational diabetes mellitus (GDM) in 2021 [3]. Preeclampsia and GDM are associated with an increase in maternal and child mortality and morbidity [4].

Insufficient vitamin D is closely associated with pregnancy-related complication, including preeclampsia and GDM [5]. However, the outcome from these studies are inconsistent, with some reviews indicate that taking vitamin D supplements did not influence the likelihood of developing preeclampsia [6],[7], while others concluding that vitamin D supplementation in pregnant women reduces the risk of preeclampsia significantly [8]–[10]. These apparently conflicting results are most likely due to high variability between studies, such as differences in dosage, form of vitamin D, timing of supplementation, and baseline conditions of pregnant women. In addition, many of the studies included had weak designs or high risk of bias, resulting in inconsistent results and, therefore, difficulty in drawing definitive conclusions [6],[7]. Furthermore, most researchers have not assessed whether the effects of supplementation differ in high-risk subgroups, such as mothers with obesity, multiparity, or a history of gestational diabetes or preeclampsia. Populations that are deficient and sufficient in vitamin D at the start of the intervention will yield different results after supplementation. Almost all researchers also did not assess whether there was an increase in 25(OH)D levels and its correlation with preeclampsia and GDM.

A review showed that vitamin D supplementation in pregnancy improved maternal and infant 25(OH)D levels, suggesting that vitamin D could influence maternal insulin resistance and fetal development [11]. Other reviews indicate that supplementing pregnant women with a combination of vitamin D and Calcium or other multi-minerals leads to a significantly lower risk of developing preeclampsia [8],[12],[13]. This review differed from our review as the researchers did not measure the effect of vitamin D supplementation only and maternal vitamin D levels to the health outcome on preeclampsia and gestational diabetes incidence.

Due to the conflicting research results, it is necessary to perform a meta-analysis on whether vitamin D supplementation reduces the preeclampsia and GDM risks and whether vitamin D deficiency during pregnancy increases the preeclampsia and GDM risks. Therefore, we aimed to evaluate the effect of vitamin D supplementation on preeclampsia and GDM incidences, as well as the effect of vitamin D deficiency on the preeclampsia, and gestational diabetes risks.

## 2. Materials and methods

This systematic review and meta-analysis complied with the Principles of Systematic Review and Meta-analysis (PRISMA) standards and the protocol has been registered with PROSPERO (CRD42024609276). A systematic search was carried out on PubMed, Scopus, and EBSCO-Medline databases to search for relevant studies around 1993 to 2025.

### 2.1. Criteria for eligibility included

Studies with cohort and randomized controlled trial designs published between 1993 and 2025. Participants were pregnant women who were given vitamin D during pregnancy (for RCT studies) or had 25(OH)D levels measured during pregnancy (for cohort studies). The primary outcome was the incidence of preeclampsia and GDM. Only original studies were included in this review, and they were restricted to articles in English. We used a combination of keywords and text words represented by ((“Vitamin D”[Mesh]) AND (((((((“Pre-Eclampsia”[Mesh]) OR (preeclampsia[Title])) OR (gestational hypertension[Title])) OR (“Diabetes, Gestational”[Mesh])) OR (“Blood Pressure”[Mesh]))))) AND ((((“pregnant women”[Title/Abstract]) OR (pregnancy[Title/Abstract])) OR (maternal[Title/Abstract])) OR (antenatal[Title/Abstract]). The key search components in this review were selected using the PICO framework to answer the research questions.

### 2.2. Study selection and data extraction

Abstracts were screened by two independent reviewers (ARS and NL). After obtaining the full text, both reviewers assessed each study according to the inclusion criteria. Any discrepancies were assessed by a third reviewer (MIK) to reach agreement. Vitamin D deficiency was defined as having a serum vitamin D concentration < 50 nmol/L (20 ng/ml) based on the American institute of Medicine (IOM) 2011 [13]. If there were studies that used multiple doses of vitamin D, the highest dose was selected for analysis.

### 2.3. Assessing the risk of bias

The study quality assessment was carried out by two reviewers (ARS and NL) using the Joanna Briggs Institute (JBI) checklist. The JBI for cohort studies included 11 assessment criteria: Similarity of the two groups from the population, exposure measured was the same for exposed and unexposed groups, exposure measured was valid and reliable, confounders identified, strategies to address confounding variables, subjects free of outcomes at the beginning of the study, outcomes measured were valid and reliable, follow-up time, follow-up completed, strategies to address drop out, and statistical analysis used. The JBI for RCT studies included 13 criteria, which were divided into 6 sections: Bias of selection and allocation, management of intervention or exposure bias, bias in assessment, measurement and detection of outcome, bias on participant retention, and statistical conclusion validity. If there was a disagreement between 2 reviewers, it was discussed with the third author.

### 2.4. Data synthesis

Cohort studies and RCT's that had sufficient data for odds ratios (OR) calculations were included in the meta-analysis. Review Manager version 5.4 (RevMan) was utilized for the statistical analysis. Results were reported in “pooled OR” or RR with 95% confidence intervals and weighted impact estimates using “forest plots”. Heterogeneity across studies was evaluated using I^2^, where heterogeneity was considered high when I^2^ > 50%. Visual examination of the “funnel plots” was conducted to evaluate potential publication bias.

## 3. Results

**Figure 1.**
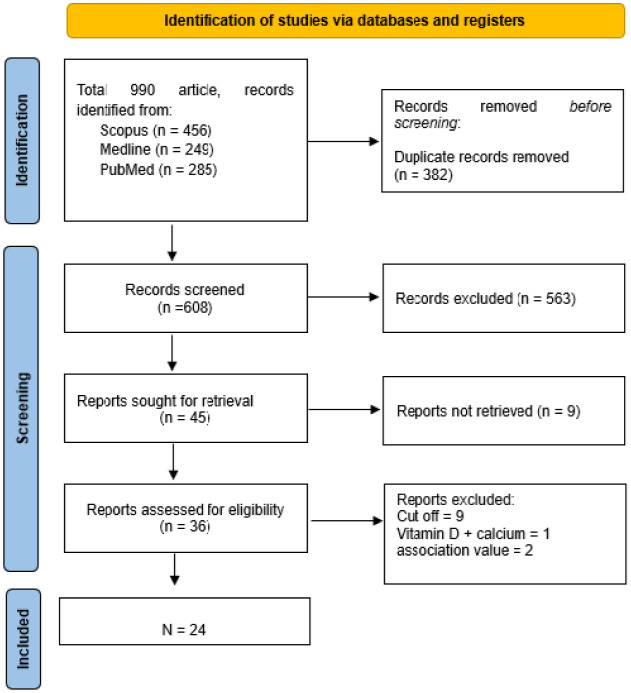
PRISMA diagram of study identification.

A total of 24 studies (8 RCT and 16 cohort) were included in this review after the screening and selection process (see Figure 1). The summary of the 24 studies, with a total of 52,372 participants, are presented in Tables 1–4.

### 3.1. Vitamin D and preeclampsia

**Figure 2.**
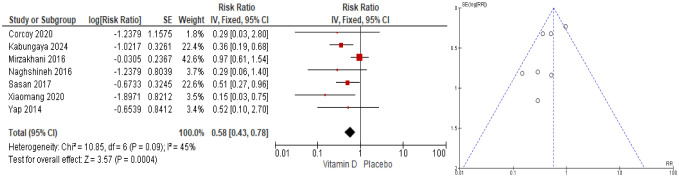
Forest and funnel plots of vitamin D supplementation on preeclampsia risk (RCTs).

In RCT studies, vitamin D supplementation during pregnancy decreased preeclampsia risk by 42% (OR = 0.58; 95% CI: 0.43–0.78; I^2^ = 45%) (Figure 2). The funnel plot shows an asymmetrical distribution of studies, with more data points concentrated on one side of the center line. This imbalance may indicate publication bias, where smaller or insignificant studies may be underrepresented. In addition, heterogeneity among studies, reflected in an I² value of 43%, indicates moderate variability. Although this level of heterogeneity is acceptable, the potential for publication bias should be considered when interpreting the overall results.

**Figure 3.**
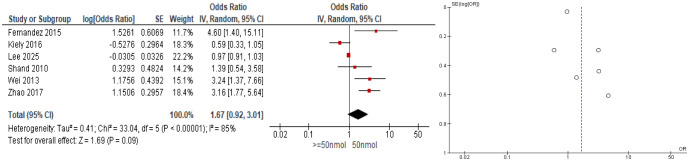
Forest and funnel plots of vitamin D deficiency effect on preeclampsia risks (cohort studies).

Meta-analysis of the cohort studies resulted in a non-significant OR of maternal vitamin D deficiency on the risk of preeclampsia (pooled OR = 1.67; 95% CI: 0.92–3.01; I^2^ = 85%). We used the cut-off of <50 nmol/L for low vitamin D concentration, which can be seen in Figure 3. The funnel plot appears asymmetrical, with studies scattered unevenly around the center line. This visual asymmetry may indicate publication bias or the small study effect, where studies with smaller sample sizes and less favorable or insignificant results may be inadequately reported or not published. Additionally, the high heterogeneity observed in the meta-analysis (I² = 85%) further indicates significant variability between studies, which may also contribute to the spread and imbalance observed in the funnel plot.

### 3.2. Vitamin D and gestational diabetes mellitus

**Figure 4.**
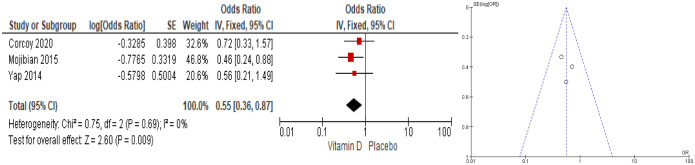
Forest and funnel plots of vitamin D supplementation effect on gestational diabetes mellitus risk (RCTs).

From RCT studies (Figure 4), it is shown that vitamin D supplementation in pregnancy decreased the GDM risk by 45% (OR = 0.55; 95% CI: 0.36–0.87; I^2^ = 0%). The timing of the intervention plays a more significant role than the dosage given. Yap's [14] study, with a dose of 5000 IU/daily beginning at the 14^th^ week until delivery did not show a significant effect on GDM with OR = 0.56 and 95% CI: 0.21–1.50, p = 0.25. Moreover, Mojibian's [15] study, with a dose of 50,000 IU every two weeks and given earlier, i.e., starting at the 12^th^ week until delivery, showed a protective effect against GDM, with an OR of 0.46, 95% CI: 0.24–0.87, p = 0.01. The heterogeneity between studies was very small (I² = 0%), indicating consistency among the included studies. The funnel plot indicated that the studies were symmetrically distributed, and there was no sign of publication bias, although the small number of studies limited the strength of this assessment. This suggests a robust finding of vitamin D supplementation's effect on GDM incidence.

**Figure 5.**
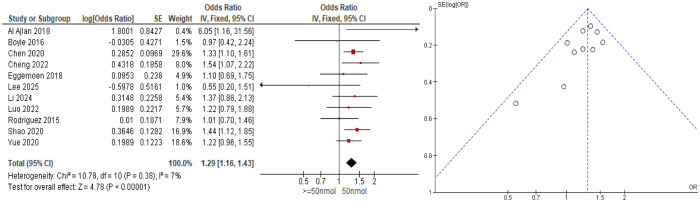
Forest and funnel plot of vitamin D deficiency in gestational diabetes mellitus (cohort studies).

To support this relationship, the cohort studies (Figure 5) suggest that low serum 25(OH)D levels (<50 nmol/L) were linked to an increase of GDM risk (OR = 1.29; 95% CI: 1.16–1.43; I^2^ = 7%) to pregnant women who had serum 25(OH)D levels of more than 50 nmol/L.

**Table 1.** Summary of RCT studies of vitamin D effect on preeclampsia.

Author, Country	Sample size	Dose	Start	End	Findings
Sasan (2017), Iran [16]	142 pregnant women who had preeclampsia history	50,000 IU vitamin D3 every two weeks	Early pregnancy	at 36^th^ week	Reduced recurrent preeclampsia, RR = 0.51 (0.27–0.98), p = 0.043
Mirzakhani (2016), United States [17]	816 pregnant women	4000 IU vitamin D daily	10^th^–18^th^ week	at delivery	No reduction in preeclampsia risk, RR = 0.97 (0.61–1.53)
Yap (2014), Australia [14]	158 pregnant women	5000 IU daily	14^th^ week	at delivery	No decrease in preeclampsia, OR = 0.51 (0.09–2.84) → RR = 0.52 (0.099–2.81)
Corcoy (2020), 7 European countries [18]	154 pregnant women	1600 IU daily	19^th^ week	at delivery	No reduction of preeclampsia, OR = 0.28 (0.03–2.79) → RR = 0.29 (0.031–2.74)
Xiaomang (2020), China [19]	407 pregnant women	4000 IU daily	13^th^ until 20^th^ week	at delivery	Reduced pre-eclampsia in 4000 IU group, RR = 0.15 (0.03–0.75), p = 0.032
Naghshineh (2016), Iran [20]	138 pregnant women	600 IU daily	16^th^ week	at delivery	No reduction of preeclampsia, RR = 0.29 (0.06–1.40), p = 0.12
Kabuyanga (2024), Congo [21]	1300 women	60,000 IU for 6 months	16^th^ week	at delivery	Reduction in preeclampsia risk, RR = 0.36 (0.19–0.69), p = 0.001

**Table 2.** Summary of cohort studies on vitamin D deficiency in preeclampsia.

Author, Country	Sample size	Time of vitamin D measurements	Findings
Wei (2013), Canada [22]	697 pregnant women	12^th^–18^th^ and 24^th^–26^th^ week	Low level at 24–26 weeks gestation increased preeclampsia risk by aOR = 3.24 (1.37–7.67), p = 0.001
Shand (2010), Canada [23]	221 pregnant women	Between 10^th^ and 20^th^ week of gestation	Low level in the initial half of gestation had no increased risk of preeclampsia, OR = 1.39 (0.54–3.53)
Kiely (2016), Ireland [24]	1768 pregnant women	15^th^ week of gestation	Low level had no significant increased risk of preeclampsia, aOR = 0.59 (0.33–1.06), but 25(OH)D concentration >75 nmol/L reduced the risk of uteroplacental dysfunction, OR = 0.64 (0.43–0.96)
Zhao (2017), China [25]	11,151 pregnant women	First, second and third trimester	Low level at 23–38 weeks increased the risk of severe preeclampsia, aOR = 3.16 (1.77–5.65), p = 0.000
Fernandez (2015), Spain [26]	257 pregnant women	First trimester (9^th^–12^th^ week)	Low level at first trimester increased the risk of preeclampsia, aOR = 4.6 (1.4–15), p = 0.010
Lee (2025), Korea [27]	5169 pregnant women	First and second trimester	Low level had no increased risk of preeclampsia, aOR = 0.97 (0.91–1.03), p = 0.295

**Table 3.** Summary of RCT studies on effect of vitamin D in gestational diabetes mellitus.

Author, Country	Sample size	Dose	Start	End	Findings
Yap (2014), Australia [14]	158 pregnant women	5000 IU daily	14^th^ week	at delivery	No reduction on the risk of GDM, OR = 0.56 (0.21–1.50), p = 0.25
Corcoy (2020), 7 European countries [18]	154 pregnant women	1600 IU daily	19^th^ week	at delivery	No reduction on GDM risk by an OR = 0.72 (0.33–1.59)
Mojibian (2015), Iran [15]	399 pregnant women	50,000 IU every 2 weeks	12^th^ week	at delivery	Reduction on GDM risk, OR = 0.46, (0.24–0.87), p = 0.01

**Table 4.** Summary of cohort studies on vitamin D deficiency effect on gestational diabetes mellitus risk.

Author, Country	Sample size	Time of vitamin D measurements	Findings
Cheng (2022), China [28]	7816 pregnant women	6^th^ to 14^th^ week of gestation	Low level increased the risk of GDM, OR = 1.54 (1.07–2.22), p = 0.019
Luo (2022), China [29]	1516 pregnant women	11^th^ to 14^th^ week of gestation	Low level did not increase the risk of GDM, aOR = 1.22 (0.79–1.88), p = 0.360
Chen (2020), China [30]	2814 mothers	≤20^th^ gestational week	Low level increased the risk of GDM, HR = 1.33 (1.100–1.618), p = 0.003
Shao (2020), China [31]	3318 pregnant women	Follow-up in the 24^th^–28^th^ week, 32^nd^–36^th^ week and 42^nd^ day postpartum	Low levels at second trimester increased the risk of GDM, OR = 1.44 (1.12–1.86), p < 0.001
Boyle (2016), New Zealand [32]	1710 women	15^th^ week of gestation	Low level did not increase the risk of GDM, aOR = 0.97 (0.42–2.25)
Al-ajlan (2018), Saudi Arabia [33]	515 pregnant women	1^st^ trimester	Low level increased the risk of GDM, aOR = 6.05 (1.16–31.42), p = 0.033
Eggemoen (2018), Norway [34]	745 pregnant women	15^th^ and 28^th^ of gestational week	Low level did not increase the risk of GDM, aOR = 1.1 (0.69–1.6), p < 0.01
Yue (2020), China [35]	8468 pregnant women	Before 20^th^ week of gestation	Low level did not increase the risk of GDM, aOR = 1.22 (0.96–1.54)
Rodriguez (2015), Spain [36]	2382 women	First trimester	Low level did not increase the risk of GDM, aRR = 1.01 (0.70–1.45)
Lee (2025), Korea [27]	5169 pregnant women	First and second trimester	Low level did not increase the risk of GDM, aOR = 0.55 (0.20–1.49), p = 0.239
Li (2024), China [37]	311 pregnant women	Second trimester	Low level did not increase the risk of GDM, RR = 1.37 (0.88–2.14), p = 0.168

## 4. Discussion

In this systematic review and meta-analysis, we examined the effect of vitamin D supplementation on preeclampsia and GDM incidences in pregnant women, and the hazard of vitamin D deficiency on preeclampsia and GDM risks. We expand the literature by combining evidence from randomized controlled trials and cohort studies. While reviews have largely focused on supplementation trials alone, this synthesis also evaluates if there is a link between vitamin D deficiency in pregnancy and a heightened risk of developing preeclampsia and GDM. By combining evidence from interventional and observational studies, our study enhances the understanding of how vitamin D influences pregnancy, highlighting not only the potential effectiveness of supplementation but also the populations that may benefit most based on baseline deficiency status.

It has been shown that vitamin D supplementation in pregnancy decreased the pre-eclampsia risk. It is suggested that this is due, in part, to the anti-inflammatory effects and intracellular signaling in calcium homeostasis of vitamin D [38],[39]. Additionally, vitamin D controls the production of adipokines linked to vascular and endothelial health [38]. Vitamin D contributes to the protection of placental blood vessel growth and the process of forming new blood vessels during the initial phase of pregnancy [40]. Another RCT study in Saudi Arabia, which was not included in this review, revealed that vitamin D supplementation of 4000IU/day decreased 16.3% of preeclampsia, but did not demonstrate a statistically significant decrease on preeclampsia risk than the low dose of 400IU/day. However, in this Saudi Arabian study, the vitamin D levels were similar in both the groups at the end of the study [41].

Vitamin D is considered crucial in pre-eclampsia development as the modulator in the immune system [42],[43]. It may facilitate maternal immune response to the placenta appropriately, thereby preventing anti-angiogenic factors' release into the bloodstream and managing hypertension [43],[44]. Active vitamin D is thought to influence the regulation of IL-10, which inhibits the expression of proinflammatory cytokines in the placenta [43].

This meta-analysis also showed that vitamin D deficiency in pregnancy did not significantly increase the pre-eclampsia risk. However, there was considerable heterogeneity among the reviewed studies. In addition, early vitamin D supplementation during pregnancy, especially in the first trimester, is linked to a reduce in preeclampsia risk. Preeclampsia is considered a disorder in the early stages of placenta formation, suggesting that adequate vitamin D intake may be necessary even earlier, possibly before embryo implantation, to have a preventive effect. Additionally, pregnant women who were deficient in vitamin D at the beginning of the intervention experienced a more significant decrease in the likelihood of developing preeclampsia. Populations with a history of preeclampsia in previous pregnancies also appear to show more beneficial effects.

The meta-analysis of cohort studies results determined that lower levels of vitamin D (<50nmol/L) was linked to a higher risk of developing preeclampsia (RR 1.67) although these results were not statistically significant, with the results and the funnel plot indicating a potential publication bias. Although we focused on vitamin D deficiency with a threshold of <50nmol/L or <20ng/ml, the Kiely study showed that 25(OH)D levels >75 nmol/L provide a protective effect against the risk of uteroplacental dysfunction as indicated by a composite outcome of SGA and pre-eclampsia [24].

In contrast, studies involving the combined use of vitamin D and calcium supplementation showed that calcium has a synergistic effect with vitamin D in reducing preeclampsia incidence [45]. Vitamin D helps increase calcium absorption and utilization in pregnant women. Calcium deficiency can cause abnormal smooth muscle contractions, resulting in increased blood pressure and a higher risk of hypertension. In the small intestine, calcium absorption is greatly dependent on vitamin D levels. In low vitamin D status, only 10%–15% of calcium from food are absorb, but in sufficient vitamin D, the absorption increases to 30%–40% [45],[46]. Thus, the availability of calcium in the diet and in the body could have important factors for the variability of the results.

An inverse relationship has been identified between plasma 1,25(OH)D and renin activity. The renin-angiotensin system (RAS) plays an important role in blood pressure regulation. During normal pregnancy, RAS is stimulated, resulting in increased circulating levels of renin, angiotensinogen, and angiotensin II. In cases of pre-eclampsia, the levels of angiotensin I, angiotensin II, and aldosterone in the bloodstream are decreased in women with normal blood pressure. Furthermore, among the preeclampsia women, active renin and autoantibodies influence the receptor of Angiotensin II type 1, which then raise the systemic blood pressure [43].

The meta-analysis in this systematic review also showed a significant risk reduction of GDM by 45% after vitamin D supplementation. Enhanced dosage level of vitamin D did not have a larger effect on GDM, but earlier intervention (at 12 weeks of pregnancy) showed more significant results compared to supplementation that began at 14 or 20 weeks. In addition, adequate vitamin D status at the start of intervention did not appear to have a significant effect on GDM. Vitamin D may work through its effect on the activity of the β-cell pancreas, which increases the production of insulin [47] and interactions with IGF signaling pathways, which improve insulin sensitivity. Some studies also showed that the vitamin D receptor (VDR) is involved in glucose metabolism regulation in both types of diabetes pathogenesis [48],[49]. Our systematic review and meta-analysis supports research that suggested insufficient vitamin D levels may be linked to a heightened risk of preeclampsia and GDM [13],[50].

Due to early pregnancy symptoms and low dietary intake, vitamin D insufficiency is more likely to occur in the first trimester than in subsequent trimesters [51].The influx of immune cells into gland cells, which causes inflammation, may be linked to functional alterations in the pancreas. Vitamin D has anti-inflammatory qualities that could help restore normal insulin production. Receptor-mediated endocytosis, which facilitates corticosteroid-dependent intracellular signaling, is facilitated by insulin receptors on peripheral cells [48]. Because vitamin D promotes renal calcium resorption and duodenal absorption, it becomes accessible for insulin-activated intracellular signaling. The regulation of glucose homeostasis may be influenced by interactions between insulin-like growth factor and the molecular components of the vitamin D cascade. Various extra-bone peripheral tissues have been found to have vitamin D receptors, which explain the vitamin's wide range of non-musculoskeletal activities, including its impact on the insulin receptor to increase insulin sensitivity. β-cells in the pancreas display VDR, which may be influenced by vitamin D [38].

Insulin resistance during pregnancy can be overcome by the release of more insulin into the blood by pancreas β-cells. This response, called β-cell compensation, is important in maintaining normal metabolism in pregnant women. β-cell compensation climaxes in the expansion of β-cell mass and augmentation. β-cell function leads to raised insulin synthesis and secretion. As a result, most mothers are protected from the development of GDM during pregnancy [47]. Active vitamin D increase insulin secretory function by β-cells. A review and meta-analysis on 2019 on 6 studies has shown the vitamin D supplementation effect during pregnancy decreases the homeostatic model assessment-insulin resistance (HOMA-IR) by about 1 level [11]. Our systematic review and meta-analysis used 3 RCT's and 9 cohort studies, and the effect was measured as GDM incidence.

Insufficient levels of vitamin D can result in heightened inflammation and reduced insulin effectiveness, potentially raising the likelihood of developing preeclampsia and GDM [39]. Vitamin D undergoes hydrolysis to 1,25(OH)D to bind to the vitamin D receptors (VDR) gland in cells, including the liver, kidney, ovarium, pituitary gland, endometrium, and pancreatic β-cells. Hydrolyzed vitamin D is useful for controlling calcium uptake in the small intestine and functions with the parathyroid hormone (PTH) to mediate bone mineralization and sustain calcium balance in the blood [39]. Vitamin D, in its active form, possesses anti-inflammatory effects and plays a role in directly initiating the transcription of insulin receptor genes. Additionally, it influences the genes' transcription related to placental invasion, proper implantation, and the angiogenesis process [39].

Vitamin D deficiency is not confined to pregnant women; it is also linked to chronic diseases, including an increased risk of cardiovascular disease [52]. In 95 patients with type 2 diabetes mellitus who had no history of coronary artery disease, vitamin D deficiency was associated with subclinical myocardial dysfunction. Decreased 25(OH)D levels were linked to impaired global longitudinal strain (GLS), p = 0.046 [53]. A study of 180 patients (80 diabetic and 60 non-diabetic) in Turkey showed that in both groups, left ventricular (LV) global longitudinal strain (GLS) was significantly impaired in patients with vitamin D deficiency (p < 0.001) compared to those without vitamin D deficiency [54].

Although the mechanisms underlying the role of vitamin D in heart disease remain unclear, their relationship is possible due to the presence of vitamin D receptors (VDR) in vascular smooth muscle cells [54]. Vitamin D is known to influence heart function by regulating the expression of the renin gene, angiotensin II, reducing left ventricular hypertrophy, and the proliferation of vascular smooth muscle cells [53],[54]. Therefore, considering the potential relationship between vitamin D deficiency and subclinical myocardial dysfunction, researchers need to evaluate the impact of early vitamin D supplementation during pregnancy on left ventricular mechanics as assessed by speckle-tracking echocardiography in pregnant women.

One of the primary strengths of our study was the inclusion of only RCT and cohort studies, which were rarely discussed together in previous reviews. Moreover, the total sample size of each group of analysis was large. Some of the other researchers used only systematic review to analyze the effect of vitamin D and preeclampsia, but we used meta-analysis and included GDM risk as the outcomes. The limitations of this review are the high degree of heterogeneity and the lack of clear reporting on baseline 25(OH)D levels in subjects included in the selected studies. This condition hinders more in-depth subgroup analysis and could potentially affect the interpretation of supplementation effectiveness. Second, research on the effects of vitamin D supplementation on GDM is limited to only 3 relevant RCT studies, which restricts our ability to draw strong conclusions. For cohort studies, there tends to be a variety of confounding factors that can potentially cause bias, which cannot be fully adjusted for in the analysis.

## 5. Conclusions

This systematic review and meta-analysis showed that vitamin D supplementation during pregnancy decreased the GDM and preeclampsia risks. Higher doses of vitamin D did not yield better effects, but supplementation in the first trimester is linked to a reduced risk of preeclampsia and GDM. Pregnant women who vitamin D deficient and had a history of preeclampsia during previous pregnancies had the greatest benefit for preeclampsia risk reduction. However, adequate vitamin D status at the start of intervention did not confer the benefit on GDM prevention. Low levels of vitamin D (<50nmol/ L) increased GDM risk, but not pre-eclampsia risk. Thus, it is recommended to give vitamin D supplementation to pregnant mothers for preeclampsia and GDM prevention, in the first trimester, especially in areas where vitamin D deficiency is prevalent.

## Use of AI tools declaration

The authors declare they have not used Artificial Intelligence (AI) tools in the creation of this article.
